# Neuron Stimulation Device Integrated with Silicon Nanowire-Based Photodetection Circuit on a Flexible Substrate

**DOI:** 10.3390/s16122035

**Published:** 2016-12-01

**Authors:** Suk Won Jung, Jong Yoon Shin, Kilwha Pi, Yong Sook Goo, Dong-il “Dan” Cho

**Affiliations:** 1 ISRC/ASRI, Department of Electrical and Computer Engineering, Seoul National University, 1 Gwanak-ro, Gwanak-gu, Seoul 08826, Korea; jungsw@keti.re.kr (S.W.J.); jshin33@snu.ac.kr (J.Y.S.); khpi1@snu.ac.kr (K.P.); 2 Human Care System Research Center, Convergence System R&D Division, Korea Electronics Technology Institute, 25 Saenari-ro, Bundang-gu, Seongnam-si, Gyeonggi-do 13509, Korea; 3 Department of Physiology, College of Medicine, Chungbuk National University, 1 Chungdae-ro, Seowon-gu, Cheongju, Chungbuk 28644, Korea; ysgoo@chungbuk.ac.kr

**Keywords:** neuron stimulation, silicon nanowire, photodetector, micro electrode, retinal prosthesis

## Abstract

This paper proposes a neural stimulation device integrated with a silicon nanowire (SiNW)-based photodetection circuit for the activation of neurons with light. The proposed device is comprised of a voltage divider and a current driver in which SiNWs are used as photodetector and field-effect transistors; it has the functions of detecting light, generating a stimulation signal in proportion to the light intensity, and transmitting the signal to a micro electrode. To show the applicability of the proposed neural stimulation device as a high-resolution retinal prosthesis system, a high-density neural stimulation device with a unit cell size of 110×110 μm and a resolution of 32×32 was fabricated on a flexible film with a thickness of approximately 50 μm. Its effectiveness as a retinal stimulation device was then evaluated using a unit cell in an in vitro animal experiment involving the retinal tissue of retinal Degeneration 1 (*rd1*) mice. Experiments wherein stimulation pulses were applied to the retinal tissues successfully demonstrate that the number of spikes in neural response signals increases in proportion to light intensity.

## 1. Introduction

Impaired connectivity or functional disorders in the neural system hinder the activation of cells or the proper transmission of information. Neural stimulation methods have been extensively investigated as potential cures for a wide range of neurological disorders, such as mental disorders, dementia, motor disabilities, and visual impairment owing to retinal degeneration [1,2,3,4]. In such investigations, electrical stimulation using extracellular electrodes is extensively used as the method for activating neurons [5,6,7]. In conjunction with the use of extracellular electrodes, methods for the activation of neurons using light are also being studied. In these methods, light-sensitive devices are used for generating electrical stimulation signals [8,9,10]. For instance, in a retinal prosthetic system, image data are acquired from external cameras connected to intraocular micro electrodes through wires [11,12,13,14] or from internal photodetectors integrated with intraocular micro electrodes [15,16,17,18]. The modulated electrical signals are then transmitted to a micro electrode array (MEA) mounted into retinal cells.

In retinal prostheses, a high-resolution system is required to provide high-quality image perception ability to a visually handicapped patient. A key to successfully developing high-resolution systems is that the stimulation signals generated from image information are individually transmitted to a large number of stimulating electrodes [19]. In the case of retinal prosthesis systems that use external cameras, such an electrical interface becomes very complicated and difficult to implement because a camera and MEA are physically distinct and separate from each other. In fact, many commercialized retinal prostheses that use an external camera, such as the Argus II system [14] from Second Sight Medical Products Inc. (Sylmar, CA, USA) or EpiRet3 [13] of EpiRet GmbH, have a low resolution; these systems have only 60 and 25 stimulating electrodes integrated into the implant device, respectively. On the other hand, in the case of retinal prostheses with embedded photodetectors for image perception within their retinal implant devices instead of using externally worn cameras, the photodetector, the signal processing circuit, and stimulating electrodes are collocated at each cell of retinal implant devices. Much research on this topic is also underway. The US Optobionics Corporation manufactured a subretinal implant device called an ‘artificial silicon retina’ (ASR) [18] and conducted clinical experiments. The ASR device comprises 5000 cells, where micro photodiodes and stimulating electrodes are integrated on a silicon substrate of approximately 25 μm in thickness. The German Retina Implant AG developed a subretinal implant device using a silicon chip, in which 1500 cells with TiN electrodes are integrated with photodiodes and amplifying circuits fabricated by a CMOS process [15]. It was also reported that, by using the above subretinal implants, patients could recognize things or letters. Furthermore, it has been found from the existing research cases that photodetectors embedded in a device can easily solve the problem of signal line connection to high-density stimulating electrodes, and thus this method is advantageous for the implementation of a high-resolution retinal prosthesis system for a high-level image perception function. Despite the recent results of various studies on the development of a high-resolution retinal prosthesis system, there still remain problems that should be solved to implement a safer and more reliable retinal prosthesis system, such as low flexibility in a retinal stimulator device, the possibility of cell necrosis due to the overheating of a retinal stimulator chip [20], and low biocompatibility of the device material [21].

As a method for light-based activation of neurons that can be applied to high-resolution retinal prosthesis systems by solving the above-described problems, this paper proposes a novel neural stimulation method as well as a flexible neural stimulation device integrated with a silicon nanowire (SiNW)-based photodetection circuit. The SiNW-based photodetection circuit comprises a voltage divider and a current driver that uses a SiNW PD [22], SiNW FETs [19], and micro electrodes. The SiNW FET and the SiNW PD are connected in series via the voltage divider, making a large resistance variation in the SiNW PD desirable because the output voltage changes when the resistance varies when light detection occurs. In the fabricated SiNW PD, the resistance is approximately 5.6 GΩ in a dark environment and approximately 2.9 MΩ at 4040 lux, corresponding to a resistance ratio of approximately 1930, which is sufficiently large and useful over a high dynamic range. Thus, the output voltage of the voltage divider at the supply voltage is 5 V and the illuminance range is 0–10,000 lux, corresponding to a very large swing range of 0.1 V (dark)–4.8 V (10,000 lux). In the current driver, where the SiNW FET and micro electrodes are connected in series, the SiNW FET is driven by the output voltage of the voltage divider and the maximum on-current at the 5 V supply voltage was found to be approximately 480 μA with an on–off ratio of $5\times 10^{4}$. Thus, the current driver has a sufficient on-current level and on–off characteristics to stimulate neurons via micro electrodes. Based on this preliminary analysis, the proposed process—stimulation signal modulation proportional to light intensity using a neuron stimulation device with a voltage divider and the current driver—could then be validated.

To demonstrate the applicability of the proposed neural stimulation method in high-resolution retinal prosthesis systems, a high-density neural stimulation device with a unit cell size of 110×110 μm and a resolution of 32×32 was fabricated on a flexible film approximately 50 μm thick. Its effectiveness as a light-based retinal stimulation device was evaluated by testing the unit cell in an in vitro animal experiment using the retinal tissue of *rd1* mice. Application of retinal stimulation pulses to the retinal tissue demonstrated that the number of spikes in neural response signals increase in proportion to light intensity.

## 2. Experimental

### 2.1. Device Design and Configuration

**Figure 1:**
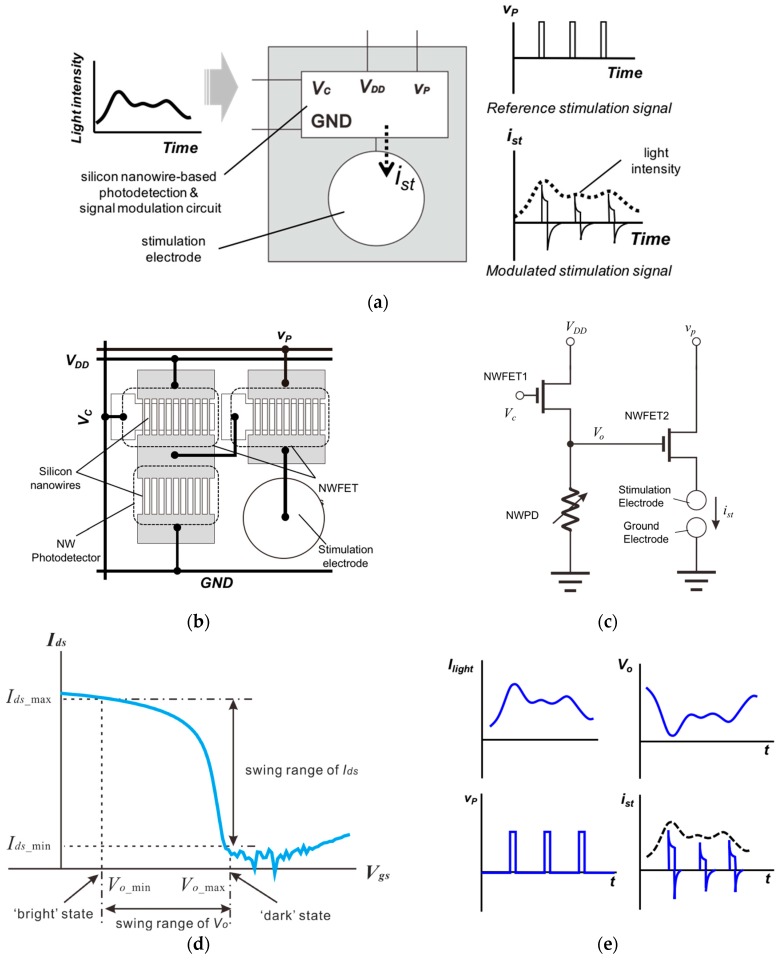

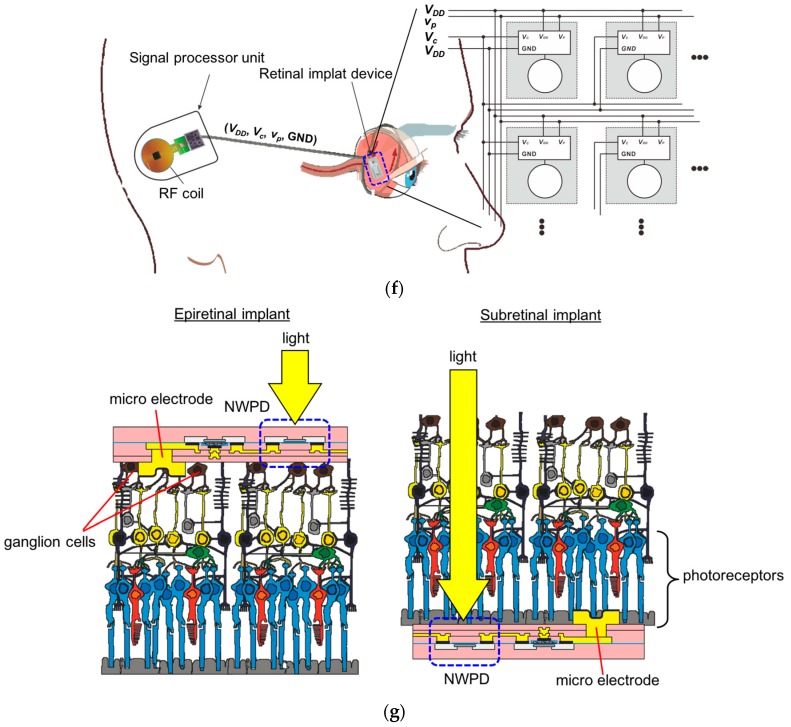
(**a**) Configuration and a block diagram of unit cell of the proposed neuron stimulation device and a modulation procedure of stimulation signal by light detection; (**b**) Configuration of device composed of SiNW FETs, a SiNW PD, and micro electrodes; (**c**) Equivalent circuit of the device; (**d**) *V_gs_-I_ds_* curve of the SiNW FET2 (NWFET2) and the relation between the *V_o_*’s swing range and the current swing range of the NWFET2; (**e**) Waveforms of the signals *I_light_*, *V_o_*, *v_p_* and *i_st_*; (**f**) Configuration of proposed device for a high resolution retinal prosthetic system and internal electrical connection diagram of the retinal prosthetic device; (**g**) Epiretinal and subretinal implantation of the proposed neural stimulation device in an eye ball.

This paper proposes a neural stimulation device and a method to evoke neural signals through the use of light. Figure 1a shows a block diagram of the unit cell of the proposed neural stimulation device and the process of neural stimulation resulting from the modulation of a stimulation signal according to the intensity of light irradiated onto the neural stimulation device. Figure 1b shows the configuration of the unit cell. The neural stimulation device comprises a SiNW PD, SiNW FETs, and micro electrodes (a stimulation electrode and a ground electrode). The resistance of the SiNW PD decreases as the light illumination intensity increases and the device can be classified as a sort of variable resistor. Thus, the neural stimulation device in Figure 1b can be expressed as the equivalent circuit in Figure 1c, which can functionally be divided into two parts: the voltage divider circuit on the left, and the current driver circuit on the right.

In the voltage divider circuit on the left, the resistance of SiNW FET1 (NWFET1) is controlled by the control voltage ($V_{c}$), and the resistance of SiNW PD (NWPD) varies with light illumination intensity. Therefore, the output voltage ($V_{o}$) of the voltage divider can be expressed by the following equation:

(1) $$V_{o}=\frac{R_{NWPD}}{R_{NWFET1}+R_{NWPD}}\cdot V_{DD},$$

where $V_{DD}$ is the supply voltage, $R_{NWFET1}$ is the resistance of NWFET1, and $R_{NWPD}$ is the resistance of NWPD. In the ‘dark’ condition with no light illumination, $V_{o}$ becomes maximum because $R_{NWPD}$ becomes maximum. As the intensity of light irradiated onto NWPD increases, both $R_{NWPD}$ and $V_{o}$ gradually decrease. If the intensity of light irradiated onto the neural stimulation device varies over time, $V_{o}$ will have a waveform with a shape inverse to the waveform of light intensity, as shown in Figure 1e.

In the current driver circuit on the right side of the equivalent circuit, NWFET2 is driven by $V_{o}$. If NWFET2 has the gate-source voltage to drain-source current ($V_{gs}-I_{ds}$) characteristics shown in Figure 1d, $I_{ds}$ becomes minimum ($I_{ds}=I_{ds\_min}$) when $V_{o}$ is maximum ($V_{o}=V_{o\_max}$, at the dark state), and $I_{ds}$ becomes maximum ($I_{ds}=I_{ds\_max}$) when $V_{o}$ is minimum ($V_{o}=V_{o\_min}$, when light intensity is the greatest). Figure 1e illustrates one example of the waveform of $v_{p}$. The reference stimulation signal of a unipolar pulse signal with fixed amplitude and frequency is applied to the drain of NWFET2. If NWFET2 is driven by $V_{o}$ with the wave waveform shown in Figure 1e, the pattern of the stimulation signal that is transmitted to the micro electrodes and neurons has the shape $i_{st}$, as shown in Figure 1e. It is seen that each pulse of the reference stimulation signal is modulated to have an amplitude proportional to light intensity; in this way, the proposed neural stimulation detects light intensity and can perform stimulation signal modulation in proportion to light intensity.

The proposed neural stimulation device can most effectively be used in retinal prosthetic systems. To do so, it is necessary to fabricate a retinal implant unit integrated with high-density cells. Figure 1f shows a method for high-density integration of the proposed neural stimulation device for application to a high-resolution retinal prosthetic system. The block diagram shows the arrangement of the device’s cells in a 2D matrix pattern and the method for the connection of signal lines for each cell. As each unit cell can modulate stimulation signals through the independent detection of light intensity, it is only necessary to connect the four signal lines $V_{DD}$, $v_{p}$, $V_{c}$, and a ground (GND) electrode. The retinal implant unit integrated with these high-density neural stimulation cells is connected to a separately fabricated signal processor. The retinal implant unit is easily implantable into the eyeball as it is fabricated in a flexible form that can be bent to fit the curvature of the eye; the signal processor unit, on the other hand, is implanted remotely. An RF coil for communication with external devices and a wireless electric power supply and a signal processing chip for controlling the implant device are embedded into the processor unit. An electrostatic discharge (ESD) protection circuit is required for a practical implementation and it can be integrated in the signal processing chip. The proposed retinal implant unit can be applied to both epiretinal and subretinal implant methods as shown in Figure 1g. In epiretinal implants, the micro electrodes are in contact with ganglion cells and the light is incident on the opposite side where the micro electrodes are formed and reaches the NWPDs. In subretinal implants, the micro electrodes are in contact with photoreceptors and the light is incident on the same side where the micro electrodes are formed and reaches the NWPDs.

### 2.2. Fabrication and Results

**Figure 2:**
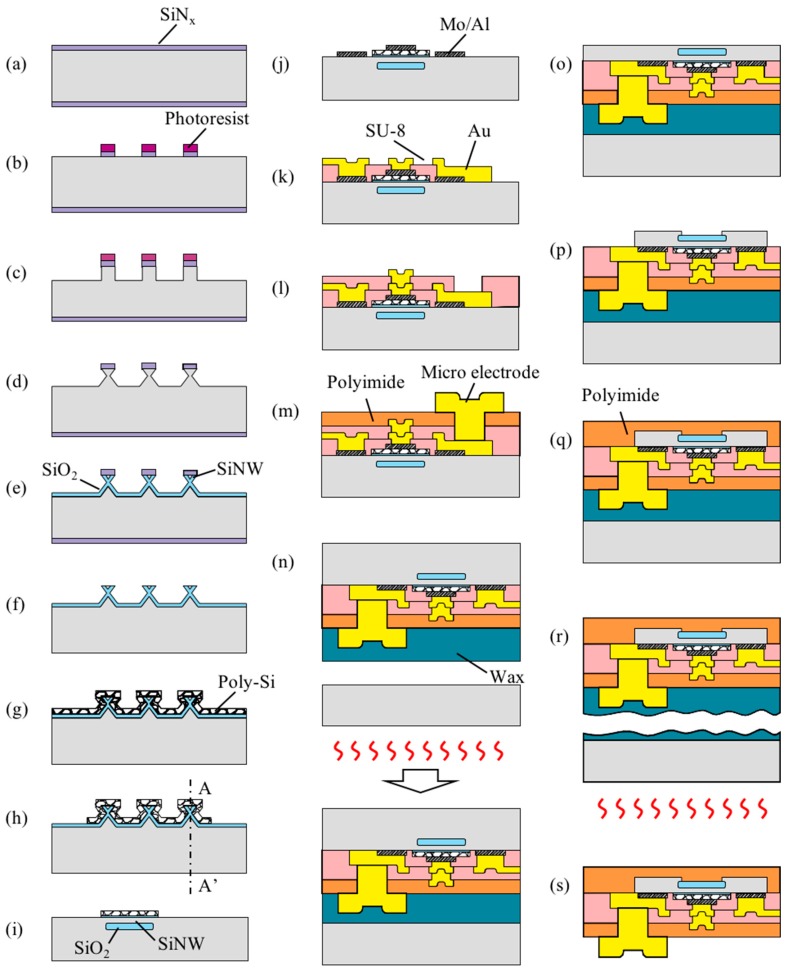
Fabrication process of the proposed high resolution neural stimulation devices integrated with SiNW-based photodetection circuit. (**a**) SiN deposition by low pressure chemical vapor deposition (LPCVD); (**b**) SiN dry etch; (**c**) Si dry etch; (**d**) Si wet etch using tetra-methyl-ammonium-hydroxide (TMAH) solution; (**e**) SiNW formation by wet oxidation process; (**f**) SiN strip by the use of H_3_PO_4_ solution; (**g**) Gate oxide and Poly-Si formation; (**h**) Poly-Si dry etch; (**i**) Cross-sectional view of A-A′ of Figure 2h; (**j**) Source, drain, gate electrodes formation; (**k**) First dielectric layer (SU-8) and electrical lines ($V_{DD}$, *GND*) formation; (**l**) Second dielectric layer and electrical lines ($V_{c}$, $v_{p}$) formation; (**m**) Third dielectric layer (polyimide) and micro electrodes formation; (**n**) Device wafer bonding on a dummy wafer; (**o**) Device wafer thinning by chemical-mechanical polishing (CMP); (**p**) Si dry etch; (**q**) Cover polyimide coating; (**r**) Separating fabricated devices from the dummy wafer; (**s**) Device cleaning by residual wax removal.

Figure 2 shows the process for fabricating the proposed neural stimulation device into a flexible form and the process for fabrication of the current driver component, in which the silicon nanowire FET and micro electrodes are connected in series. In the first step, silicon nanowires are formed using the top-down method (Figure 2a–e) [23]. Subsequently, polysilicon gates and source and drain electrodes are fabricated (Figure 2f–j). The electric signal lines ($V_{DD}$, $v_{p}$, $V_{c}$ and GND) are then formed via a multi-layer metallization process (Figure 2k,l). A 5 μm-thick polyimide layer is patterned and Au micro electrodes are then formed via an Au electroplating process (Figure 2m). For biocompatibility, a Pt-black layer can be formed on the surface of the Au electrodes by a successive Pt electroplating process after the Au electroplating process [24]. Theoretically, TiN micro electrodes can be fabricated by TiN sputtering and RIE (Reactive Ion Etching) processes instead of electroplating. In this paper, Pt-black micro electrodes are fabricated and are used for the following in vitro experiments. After the micro electrodes are fabricated, the device wafer is bonded with a dummy wafer using a bonding wax, BGL-7160 (AI Technology Inc., Princeton, NJ, USA), and the device wafer is thinned to approximately 30 μm via a chemical-mechanical planarization (CMP) process (Figure 2n,o). Photoresist (PR) patterning is performed and the silicon is etched by using a deep reactive ion etch (DRIE) process (Figure 2p). The bonding wax is melted by heating the substrate to 160 °C, and the fabrication of the flexible neural stimulation device is completed by separating the neural stimulation device from the dummy substrate (Figure 2q–s).

**Figure 3:**
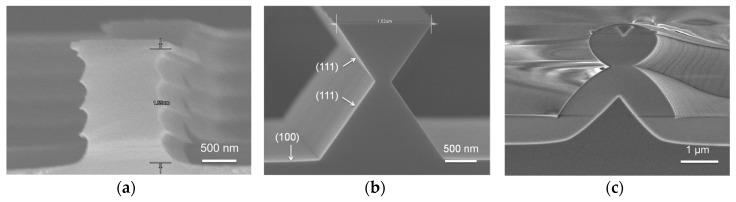
Fabricated SiNWs. (**a**) Formation of column structures by Si deep RIE process; (**b**) Formation of hourglass shaped structures by Si anisotropic wet etch process; (**c**) SiNW formation by wet oxidation process.

**Figure 4:**
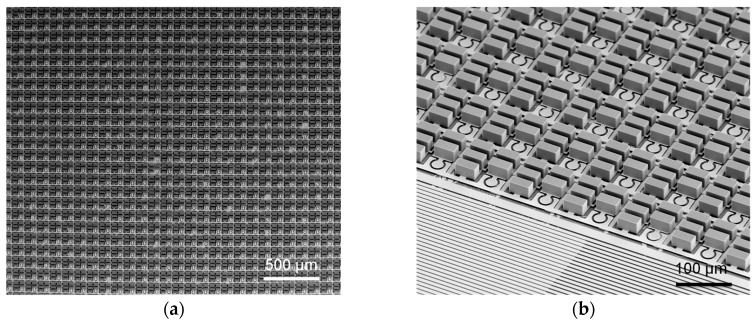

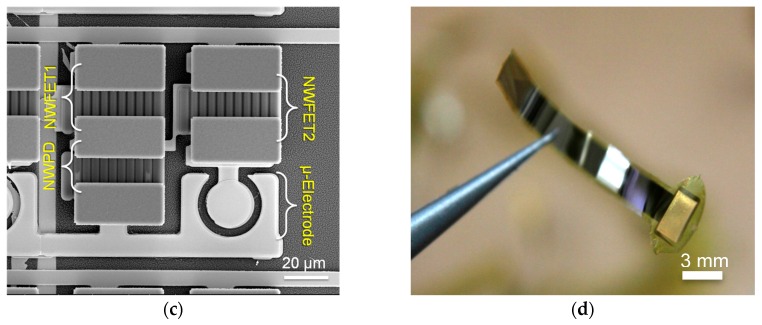
Fabricated high-resolution neural stimulation device. (**a**) Top view of the fabricated device after Si deep RIE process of Figure 2p; (**b**) Perspective view of Figure 4a; (**c**) Unit cell of the device; (**d**) Flexible neural stimulation device with 32 × 32 resolution.

Figure 3a shows the shape of the column structure after the dry etching of silicon in Figure 2c: scallops formed on the sidewall of the structure are seen, as the Si DRIE process is used. As seen in Figure 3b, following wet etching using tetra-methyl-ammonium-hydroxide (TMAH) solution, the scallops disappear as the sidewall of the column structure is etched. Figure 3c shows the triangular shape of a silicon nanowire formed following the wet oxidation process. It is seen that the top of the silicon nanowire has formed with a width of approximately 450 nm. Figure 4 shows the 32×32 neural stimulation device fabricated using the process in Figure 2. Figure 4a–c show the device after the Si DRIE process in Figure 2p has been completed. The size of the unit cell is 110×110 μm and it used 10 SiNWs with a length of 20 μm each to compose NWFET1, NWFEET2, and NWPD. A center microelectrode 30 μm in diameter with a surrounding ground electrode is also present. Figure 4d shows the neural stimulation device separated from the substrate after the process has been completed. The device is approximately 50 μm thick and it is very flexible.

The characteristics of NWPD and NWFET depend on the thickness and doping concentration of the silicon nanowires. In this paper, the thickness of silicon nanowires is controlled by a photolithography process, RIE processes, a Si TMAH wet etching process, and an oxidation process. In the case of TMAH silicon wet etching process, even though there is a slight difference in the wet etching time, an etch stop condition is formed by the hourglass silicon structure so that the deviation of the top width of the silicon structure is insignificant. In the remaining processes, the process deviations are controlled to within approximately 5%, and therefore, the effects on the thickness of the nanowire are not significant. The doping concentration of the silicon nanowires can also be precisely controlled by an ion implantation process. Therefore, the influences of the process variation on the characteristics of NWPD and NWFET are not significant.

## 3. Results and Discussions

### 3.1. Device Characterization

**Figure 5:**
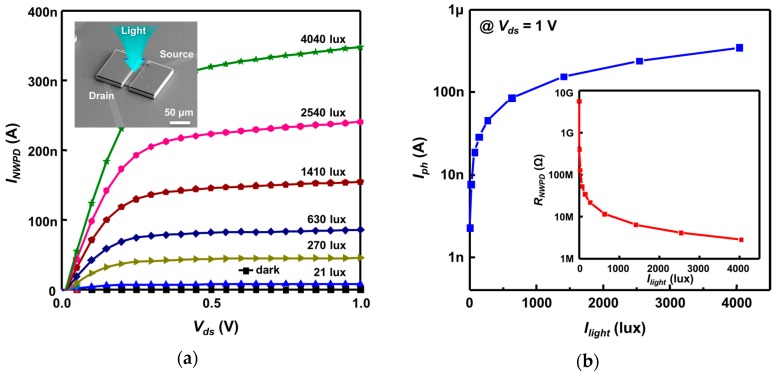

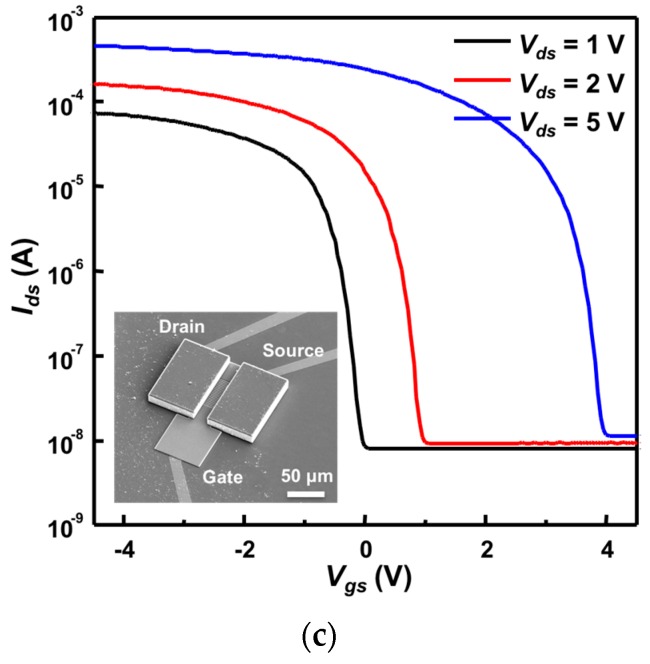
(**a**) *I*-*V* curves of the fabricated SiNW PD against varying light illumination intensity; (**b**) Photocurrent and resistance values of the SiNW PD against light intensity; (**c**) $V_{gs}-I_{ds}\;$ curves of the SiNW FET for several bias voltages.

SiNW PDs and SiNW FETs, the basic devices composing the neural stimulation device, were fabricated using the process shown in Figure 2, following which the characteristics of each device were examined. To investigate the resistance variation of an SiNW PD as a function of light intensity, the photo-induced output ($I_{ds}\sim V_{ds}$) characteristics of the SiNW PD were measured at varying halogen lamp-produced light intensities ($I_{light}$), as shown in Figure 5a. Figure 5b shows the results of calculating the photocurrent ($I_{ph}$) and resistance ($R_{NWPD}$) of the SiNW PD against light intensity for $V_{ds}$ = 1 V using the *I*–*V* graph in Figure 5a. The graph shows that both $I_{ph}$ and $R_{NWPD}$ abruptly change within an illuminance range of low light intensity, above which the change is more gradual. $R_{NWPD}$ shows a high value of approximately 5.6 GΩ in a dark environment and approximately 2.9 MΩ in a bright environment of 4040 lux. Thus, the resistance change rate is so great that the ratio between the two resistance values, $R_{NWPD\left(dark\right)}/R_{NWPD\left(bright\right)}$, goes as high as 1930. In particular, it is apparent from the graph that the SiNW PD shows high sensitivity in a low illuminance range.

The relative resistance of the SiNW FET used in the voltage divider circuit to that of the SiNW PD is important. The resistance of the SiNW FET $(R_{NWFET}$) is controlled by the gate-source voltage ($V_{gs}$), and the value that it can obtain is shown in Figure 5c, which shows the transfer ($I_{ds}\sim V_{gs}$) characteristics of a fabricated SiNW FET. If the conditions of $R_{NWPD\left(dark\right)}\gg R_{NWFET}$ in a dark environment and $R_{NWPD\left(bright\right)}\ll R_{NWFET}$ in a bright environment are satisfied, the voltage range $V_{o}$ of the voltage divider is found according to Equation (1) to be $V_{DD}$ (dark)–0 V (bright), resulting in the maximum swing range. For this, $V_{gs}$ must be set so that $R_{NWPD\left(dark\right)}\gg R_{NWFET}\gg R_{NWPD\left(bright\right)}$. On the other hand, the threshold voltage ($V_{th}$), maximum on-current ($I_{ON\_max}$), and on–off ratio are important parameters for the SiNW FET used in the current driver circuit. In Figure 5c, the threshold voltage moves from approximately 0 V (when $V_{ds}$ is 1 V) to 4 V (when $V_{ds}$ is 5 V). $I_{ON\_max}$ is approximately 480 μA when $V_{ds}$ is 5 V, and the on–off ratio of SiNW FET is approximately $5\times 10^{4}$ when $V_{ds}$ is 5 V. Thus, it is found that the SiNW FET has sufficient on-current level and on–off characteristics for use as the current driver circuit.

**Figure 6:**
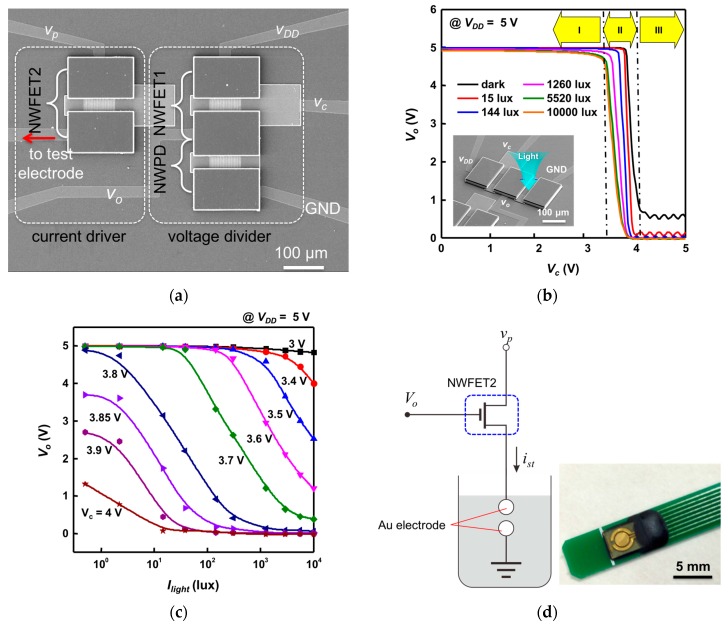

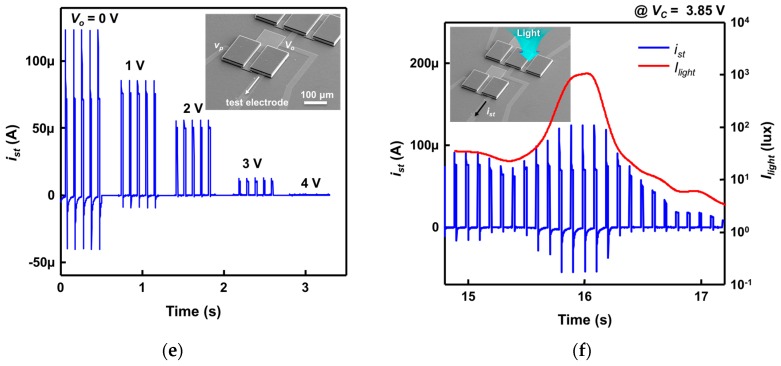
(**a**) Fabricated neural stimulation device; (**b**) Output voltage curve of the voltage divider against control voltage by light intensity; (**c**) Output voltage curve of the voltage divider against light intensity by control voltage; (**d**) Measurement setup of the current driver; (**e**) Waveform of current is transmitted to the micro electrode against gate voltage $V_{o}$ when $V_{c}$ = 3.85 V; (**f**) Modulation waveform of stimulation signal for the neural stimulation device, which is proportional to light intensity.

Figure 6 shows the results of an experiment evaluating the characteristics of both the voltage divider and current driver (which together form the proposed neural stimulation device) and verifying the previously discussed process of stimulation signal modulation. Figure 6a shows the fabricated neural stimulation device. Figure 6b shows the measurement results obtained from the voltage divider, in which $V_{o}$ was measured against $V_{c}$ by varying the intensity of light irradiated onto the SiNW PD. In Figure 6b, it is seen that if $V_{c}<3.4\;V$ (Region I in Figure 6b), $V_{o}$ remains at close to $V_{DD}$ = 5 V despite the variations in $R_{NWPD}$ induced by variations in light intensity. This occurs because the value of $V_{c}$ that determines that $R_{NWFET1}$ always remains much smaller than $R_{NWFET1}$ even though $R_{NWPD}$ is lowered under a bright environment: that is, $R_{NWFET1}\ll R_{NWPD}$. Under this condition, Equation (1) gives $V_{o}\approx V_{DD}$ regardless of the light intensity. By contrast, if $V_{c}>4.1\;V$ (Region III in Figure 6b), $V_{o}$ remains in the very low range of 0–0.6 V despite variations in light intensity. This is a case that falls under the condition in which $R_{NWFET1}\gg R_{NWPD}$, under which Equation (1) gives $V_{o}\approx 0$ V regardless of the light intensity. For the region $3.4\;V<V_{c}<4.1\;V$ (Region II in Figure 6b), the output characteristics of $V_{o}$ are shown in the graph in Figure 6c, in which the *x*-axis is replaced by light intensity. If $V_{c}$ is set close to 3.8 V, the ideal condition of the voltage divider, $R_{NWPD\left(dark\right)}\gg R_{NWFET}\gg R_{NWPD\left(bright\right)}$, is satisfied. Therefore, when $V_{c}$ is 3.8 V, $V_{o}$ has the maximum output range of 4.8 V (dark) to 0.1 V (10,000 lux). From the graph, it is apparent that for $V_{c}$ = 3.8 V and above, the voltage divider works sensitively in the range of low illuminance below 100 lux and is increasingly insensitive as illuminance increases. By contrast, for $V_{c}$ = 3.8 V and below, it is seen that the voltage divider is rather insensitive in the range of low illuminance and works sensitively in the range of high illuminance above 100 lux. In addition, as $V_{o}$ acts as the gate voltage of the NWFET2 of the current driver circuit that determines the output current level, it is necessary to consider the light intensity range and the level of concomitant output current in the selection of the value of $V_{c}$. Figure 6d shows an experimental setup for investigating the output characteristics of the current driver circuit. The drain of NWFET2 is connected to a reference stimulation signal generated by a function generator having an amplitude of 5 V and a duration of 20 ms. The reference stimulation signal used in the experiment is monophasic, but a biphasic pulse signal can also be used. The drain of NWFET2 is connected to the reference stimulation signal generated by a function generator. The reference stimulation signal has an amplitude of 5 V and a duration of 20 ms. The source of NWFET2 is connected to the Au test electrode, and the test electrode is immersed in phosphate buffered saline (PBS) solution. Figure 6e shows the waveform of current transmitted to the micro electrode measured against gate voltage ($V_{o}$). The maximum peak current for $V_{o}$ is approximately 123 μA when $V_{o}$ is 0 V, and when $V_{o}>4\;V$, NWFET2 is off and therefore the current signal is no longer transmitted. Figure 6f shows the waveform of a stimulation current signal ($i_{st}$) transmitted to test electrodes after randomly varying the intensity of light illuminated onto a neural stimulation device employing a voltage divider and a current driver. The illuminance waveform ($I_{light}$) and the concomitant stimulation current waveform are simultaneously depicted on the graph. It is seen that each pulse of the reference stimulation signal is modulated to have an amplitude that is relatively proportional to light intensity. The above results show that the proposed neural stimulation device detects light intensity and can perform stimulation signal modulation in proportion to the light intensity.

### 3.2. In Vitro Experiment

**Figure 7:**
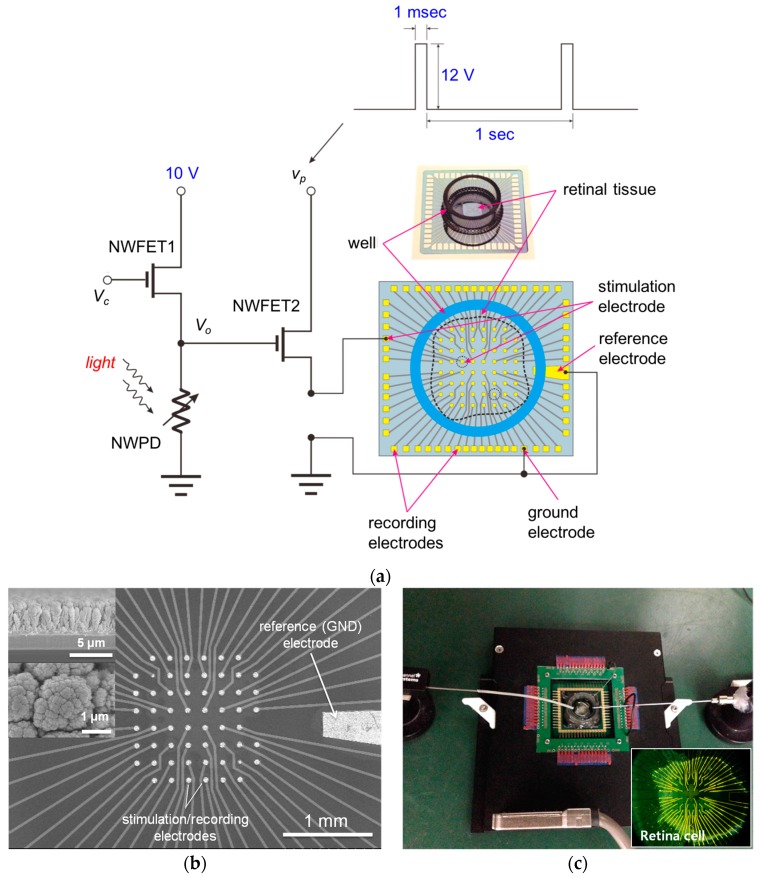
(**a**) Circuit diagram of in vitro animal experimental setup; (**b**) Micro electrode array for recording multi-channel neural signals; (**c**) Arrangement of micro electrode array and retinal tissue using multi-channel probing system (in vitro MEA-Systems of Multi Channel Systems).

An in vitro animal experiment was performed in order to evaluate the effectiveness of activating neurons using the fabricated neural stimulation device. The experimental setup was configured as shown in Figure 7a. A drain voltage ($V_{DD}$) of 10 V was applied to the voltage divider of the neural stimulation device, and a uniform rectangular pulse $v_{st}$, with amplitude 12 V, pulse width (duration) 1 ms, and frequency 1 Hz, was generated using a field programmable gate array (FPGA). The source side of NWFET2 was connected to one of the MEA electrodes, as shown in Figure 7a. The MEA, which is shown in Figure 7b, comprised 60 micro electrodes and a reference electrode. The micro electrode was a circular electrode 50 μm in diameter and was fabricated by forming an Au (~2 μm) layer and a Pt-black layer (~4 μm) via successive electroforming processes. The Pt-black layer of the micro electrode had a wide surface area owing to its nano-porous structure and had the effects of lowering the interface impedance between the micro electrodes and the retinal cells and enlarging the injection charge capacity [24]. One MEA electrode connected to the current driver was used to stimulate the retinal nerve, and the remaining electrodes were used to record neural response signals. The retinal cell for the in vitro experiment was extirpated from an *rd1* mouse. As shown in Figure 7c, the separated retinal cell was stuck closely to the MEA chip so that the ganglion cell could come into contact with the MEA. A well structure was formed around the retinal cell and then filled with cerebrospinal fluid (CSF) so that the retinal cell could maintain its function for a long time.

**Figure 8:**
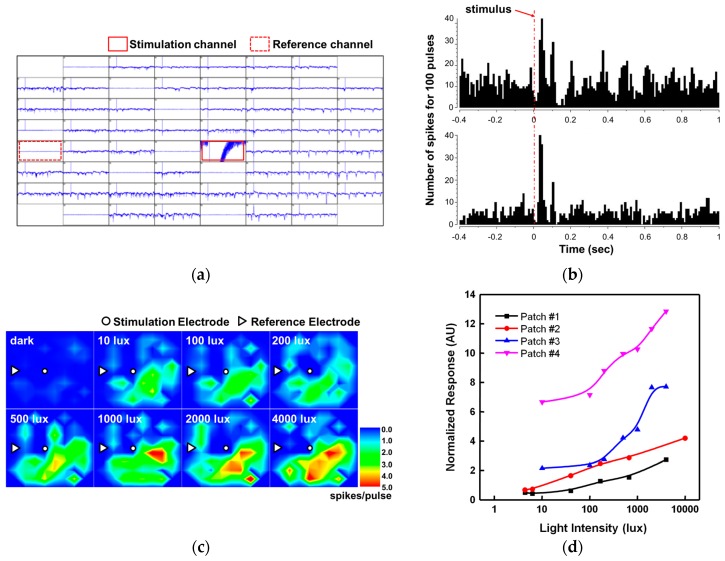
(**a**) Neural response signals recorded at 60 channels; (**b**) Changes in the number of spikes during, before, and after the application of stimulation signals; (**c**) Number of spikes per pulse at each channel against light intensity; (**d**) Normalized response by light intensity as a result of stimulation experiment using four retinal tissues.

Figure 8a–d shows the neural response signals produced in the in vitro experiment using the neural stimulation device. Figure 8a shows an example of the neural response signals at 60 channels and represents the locations of a stimulating electrode and of a reference electrode. The effectiveness of stimulation can be evaluated by observing significant changes in the number of spikes after the stimulation signal is applied. Figure 8b shows the results of analyzing the neural response signals of two channels. The histogram graph represents the total number of spikes in neural signals in an interval of 10 ms before and after the application of 100 stimulation pulses. In Figure 8b, a distinctive increase in the number of spikes can be identified within about 0.2 s after application of the stimulation pulses. Figure 8c is a color map that represents the number of spikes per pulse (spikes/pulse) obtained from the histogram of Figure 8b against light intensity for 60 channels. As is seen in Figure 8c, the number of spikes increase prominently as the light intensity increases. The neural response against intensity of stimulus can be quantified using the normalized response defined by Equation (2).

(2) $$\mathrm{normalized}\;\mathrm{response}=\;\frac{\sum_{\mathrm{ch}}\mathrm{spikes}\;\mathrm{per}\;\mathrm{pulse}\;\mathrm{after}\;\mathrm{stimulation}}{\sum_{\mathrm{ch}}\mathrm{spikes}\;\mathrm{per}\;\mathrm{pulse}\;\mathrm{before}\;\mathrm{stimulation}},$$

Figure 8d shows the normalized response curves for four patches of retinal tissue extirpated from *rd1* mice. It is seen that the retinal neurons tend to respond in proportion to the amplitude of the stimulation pulse (i.e., the intensity of light) although there exist some differences in level and shape among the normalized response curves.

## 4. Conclusions

This paper proposed a method for neural stimulation and a neural stimulation device integrated with an SiNW-based photodetection circuit. The SiNW-based photodetection circuit is designed to have the functions of detecting light and generating a stimulation signal in proportion to light intensity that is then transmitted to a micro electrode. Large resistance variation in the SiNW PD of the voltage divider circuit of a neuron stimulation device is desirable, and the fabricated SiNW PD produces a very large ratio $R_{NWPD\left(dark\right)}/R_{NWPD\left(bright\right)}$ of 1930. Owing to the large resistance variation of the SiNW PD, the voltage divider exhibits a very large swing range of output voltage. In the current driver circuit of the neuron stimulation device, the SiNW FET, which is driven by the output voltage of the voltage divider, produces a maximum on-current of approximately 480 μA and an on–off ratio of $5\times 10^{4}$. It was determined that the current driver has a high enough current value and sufficient on–off characteristics to stimulate neurons via micro electrodes. The proposed neuron stimulation device can be applied to a high-resolution retinal prosthesis system, as was demonstrated by fabricating a high-density neural stimulation device with a unit cell size of 110×110 μm and a resolution of 32×32 in the form of a flexible film about 50 μm thick. Its effectiveness as a retinal stimulation device was evaluated by utilizing a unit cell of the neural stimulation device in an in vitro animal experiment involving the retinal tissue of *rd1* mice. A reference stimulation signal of uniform rectangular pulses modulated by the neural stimulation device into a stimulation signal proportional to light intensity was produced and transmitted into micro electrodes to stimulate the retinal nerve. Application of the retinal stimulation pulses to the retinal tissues demonstrated that the number of spikes in neural response signals increase in proportion to light intensity. These results address new challenges in developing neuron stimulation devices using SiNW-based circuits and are a promising step in the development of high-resolution retinal prosthetic systems.
